# The Occupational Health and Safety of Sign Language Interpreters Working Remotely During the COVID-19 Pandemic

**DOI:** 10.5888/pcd19.210462

**Published:** 2022-06-09

**Authors:** Gretchen Roman, Vincent Samar, Deborah Ossip, Michael McKee, Steven Barnett, Reza Yousefi-Nooraie

**Affiliations:** 1 Clinical and Translational Science Institute, University of Rochester, Rochester, New York; 2 Department of Public Health Sciences, University of Rochester, Rochester, New York; 3 Department of Liberal Studies, National Technical Institute for the Deaf, Rochester Institute of Technology, Rochester, New York; 4 Department of Family Medicine, University of Michigan, Ann Arbor, Michigan; 5 Department of Family Medicine, University of Rochester, Rochester, New York

## Abstract

**Introduction:**

The COVID-19 pandemic has caused a dramatic shift in work conditions, bringing increased attention to the occupational health of remote workers. We aimed to investigate the physical and mental health of sign language interpreters working remotely from home because of the pandemic.

**Methods:**

We measured the physical and mental health of certified interpreters who worked remotely 10 or more hours per week. We evaluated associations within the overall sample and compared separate generalized linear models across primary interpreting settings and platforms. We hypothesized that physical health would be correlated with mental health and that differences across settings would exist.

**Results:**

We recruited 120 interpreters to participate. We calculated scores for disability (mean score, 13.93 [standard error of the mean (SEM), 1.43] of 100), work disability (mean score, 10.86 [SEM, 1.59] of 100), and pain (mean score, 3.53 [SEM, 0.29] of 10). Shoulder pain was most prevalent (27.5%). Respondents had scores that were not within normal limits for depression (22.5%), anxiety (16.7%), and stress (24.2%). Although disability was not associated with depression, all other outcomes for physical health were correlated with mental health (*r *≥ 0.223, *P* ≤ .02). Educational and community/freelance interpreters trended toward greater adverse physical health, whereas educational and video remote interpreters trended toward more mental health concerns.

**Conclusion:**

Maintaining the occupational health of sign language interpreters is critical for addressing the language barriers that have resulted in health inequities for deaf communities. Associations of disability, work disability, and pain with mental health warrant a holistic approach in the clinical treatment and research of these essential workers.

**Summary**

**What is known on this topic?**

The occupational health risks and associations between physical and mental health in sign language interpreters working onsite or face-to-face have been well supported; however, the effects of working remotely from home remain unknown.

**What is added by this report?**

We investigated the occupational health of sign language interpreters working remotely from home because of the COVID-19 pandemic. Although we found no significant differences across interpreting settings, this study served a heuristic purpose in descriptively identifying trends to guide further research with larger sample sizes.

**What are the implications for public health practice?**

Physical health associations with mental health warrant a more holistic approach in the clinical treatment and research of these essential workers.

## Introduction

The US Bureau of Labor Statistics implemented a supplement to the Current Population Survey to measure the effects of the COVID-19 pandemic on the labor market. The earliest available data from May 2020 demonstrated that 35.4% of employed persons teleworked from home because of the pandemic (1). This teleworking varied based on occupation. In May 2020, sign language interpreters were among the 70.2% of those in community and social service occupations working from home because of the pandemic. By December 2021, 19.7% of these workers continued to work from home (1).

The occupational health risks and associations between physical and mental health in sign language interpreters working onsite or face-to-face have been well supported (2–4); however, the effects of working remotely from home remain unknown. Before the pandemic, an increasing trend for sign language interpreters to provide prescheduled and on-demand video remote interpreting (2-way connection between onsite participants and a remote interpreter in a separate location) was emerging (5). Research has explored video remote interpreting across conference, medical, legal, mental health, and educational settings and emphasized the importance of best teaching practices in interpreter education programs (6). Tyer found that intermittent face-to-face work helped to mitigate the loneliness and professional isolation expressed by interpreters when teleworking (5). However, because this research focused exclusively on video remote interpreting, further investigation is needed on the effects of working remotely from home across interpreting settings and platforms.

Remote interpreting is “any form of simultaneous interpreting where the interpreter works away from the meeting room, either through a video-conferencing set-up or through a cabled arrangement close to the meeting facilities, either in the same building or at a neighboring location” (7). To the best of our knowledge, past studies on remote interpreting were conducted with spoken language interpreters (8,9), and limited work investigated sign language interpreters working remotely from home during the COVID-19 pandemic. Schnack found that 30% of sign language interpreters working from home across video remote and video relay (3-way connection over the telephone) interpreting settings during the pandemic had mild to moderate anxiety, 10% experienced extremely severe stress levels, and 80% did not feel connected to colleagues (10). De Meulder et al found that 67% of sign language interpreters working from home across educational (ie, K–12 or postsecondary), staff (ie, agency, government, business, technology, or hospital employee), and video relay settings during COVID-19 agreed that remote interpreting was more stressful than onsite interpreting (11). Respondents expressed that remote interpreting was physically difficult, and 13% sought mental health support as a result of the changing professional demands of the pandemic (11). Further exploration of the occupational health concerns of interpreters working remotely, including additional measures of physical and mental health, will complement these recent works.

Sign language interpreters are critical for ensuring equitable health care access, appropriate health care use, and completion of protective preventive care among deaf sign language users (12,13). There is heightened concern about how changes in work conditions may affect the physical and mental health of sign language interpreters because such changes may exacerbate health inequities in deaf communities. The objective of this research was to investigate the occupational health of sign language interpreters working remotely from home because of the COVID-19 pandemic. Our primary analyses focused on measuring the physical and mental health of interpreters while working remotely, as well as determining associations between outcome variables. Our secondary analyses compared the outcome variables across staff, educational, community/freelance (ie, independent contractor), video remote, and video relay interpreting settings. We hypothesized that the physical health of sign language interpreters working remotely would be positively correlated with mental health and that differences in the outcome variables would be realized across interpreting settings.

## Methods

### Participants

We recruited certified sign language interpreters who were bilingual in English and American Sign Language. Stakeholders at prospective nonprofit associations, educational institutions, video relay service providers, and interpreter referral services were asked to advertise this study by sharing provided recruitment material via email listservs, social media, or websites. Adults aged 18 years or older were eligible to participate if they worked remotely as sign language interpreters 10 or more hours per week and had been certified by the Registry of Interpreters for the Deaf, National Association of the Deaf, interpreter’s state of residence, or Board for Evaluation of Interpreters Certification Program.

Little work on the physical and mental health of interpreters working remotely since the start of the COVID-19 pandemic was available to estimate sample size for this study. Roziner and Shlesinger provided dependent mean differences (matched pairs) in spoken language interpreters’ (N = 30) perception of stress between onsite and remote interpreting. We used a paired samples *t* test statistic (*P* < .01) on the need for recovery from stress measure (9) to manually compute their effect size (Cohen *d* = 0.59). We conducted a power analysis (*d* = 0.59; α = .05; power = 0.80; numerator degrees of freedom = 4; groups = 5; covariates = 1) using G*Power 3.1.9.7 software to estimate the total number of participants (N = 40) and number of participants per interpreting setting (n = 8). We aimed to recruit a larger total sample size to increase the likelihood of achieving significance across settings.

This cross-sectional study was reviewed by the University of Rochester’s Research Subjects Review Board (no. STUDY00005893) and was deemed exempt.

### Data collection

From March through September 2021, all interested participants accessed a link to a collective online survey instrument (REDCap). Interpreters identified their primary and, if applicable, secondary and tertiary interpreting settings.

We measured physical health by obtaining disability and work disability scores from the shortened version of the Disabilities of the Arm, Shoulder and Hand (QuickDASH) and the optional work module (DASHWork), as well as the Numeric Pain Rating Scale (NPRS) (14,15). Overall score and depression, anxiety, and stress subscale scores on the shortened version of the Depression, Anxiety, and Stress Scale (DASS-21) were used to measure mental health (Table 1) (16).

**Table 1:** Description of Tools Used to Measure Physical and Mental Health of Sign Language Interpreters in the US, Canada, and England Working Remotely During the COVID-19 Pandemic (N = 120), Rochester, New York, March–September 2021

Outcome variable	Tool	Description
Physical health	QuickDASH (14)	The QuickDASH has 5 sections with a total of 11 questions. Participants completed the QuickDASH, along with 4 additional items from the optional work module (DASHWork). Using a 5-point Likert scale, the QuickDASH produces a disability score and the DASHWork produces a work disability score, with 0 indicating no disability and 100 equating to maximum disability. However, item ratings vary (1 = no difficulty to 5 = unable; 1 = not at all to 5 = extremely; 1 = not limited at all to 5 = unable; 1 = none to 5 = extreme; 1 = no difficulty to 5 = so much difficulty that I can’t sleep).
	NPRS (15)	Participants disclosed whether they experience musculoskeletal pain while interpreting remotely and if so, in what body region. If more than 1 region was identified, the primary region experiencing pain was noted. Pain intensity of the primary body region was measured by using the NPRS (0 = no pain to 10 = the worst imaginable pain).
Mental health	DASS-21 (16)	The DASS-21 has depression, anxiety, and stress subscales, each with 7 items for a total of 21 questions. Ratings for each item (0 = never to 3 = almost always) are summed and multiplied by 2. Higher-rated responses indicate worse depression, anxiety, or stress, with maximum scores of 42 on each subscale and 126 on the total measure. Normal depression, anxiety, and stress scores can range from 0–9, 0–7, and 0–14, respectively, while normal levels for the overall DASS-21 can range from 0–30. For depression, scores of 10–13, 14–20, 21–27, and ≥28 equate to mild, moderate, severe, and extremely severe depression, respectively. For anxiety, a score of 8–9 is considered mild, 10–14 moderate, 15–19 severe, and ≥20 extremely severe. Lastly, for stress, a score of 15–18 indicates mild, 19–25 moderate, 26–33 severe, and ≥34 extremely severe levels of stress (17).

Abbreviations: DASHWork, optional work module of QuickDASH; DASS-21, Depression, Anxiety, and Stress Scale; NPRS, Numeric Pain Rating Scale; QuickDASH, shortened version of the Disabilities of the Arm, Shoulder, and Hand.

### Data analysis

We calculated descriptive statistics (mean and standard error of the mean [SEM]) for patient demographics, QuickDASH, DASHWork, NPRS, and DASS-21 and compared mental health measures with normative values (17). The levels of categorical demographics were collapsed to reduce the number of degrees of freedom for purposes of the analysis (sex was coded to male, female, and other; hearing status was eliminated because of the low frequency across levels; race was coded to White/non-Hispanic and other; and education was coded to a 2-level covariate of high school diploma or less and some college or more). We evaluated differences across primary interpreting settings for the categorical (ie, sex, hearing status, race, and education) and continuous (ie, age) covariates using Pearson χ^2^ tests and a Kruskal–Wallis test, respectively (Table 2). We used a Spearman rank correlation coefficient (ρ) to measure associations within the overall sample between outcomes for physical and mental health. While adjusting for age, separate generalized linear models using a scale response of gamma distribution with log link evaluated differences across primary settings for QuickDASH, DASHWork, NPRS, overall DASS-21, and DASS-21 depression, anxiety, and stress. Participants who identified their primary setting as “other” were not included in the secondary analyses. All statistical analyses were performed by using SPSS version 27 (IBM Corp), and significance was set at *P* < .05.

**Table 2:** Demographic Characteristics of Participants (N = 120), Study of the Physical and Mental Health of Sign Language Interpreters in the US, Canada, and England Working Remotely During the COVID-19 Pandemic, Rochester, New York, March–September 2021^a^

Characteristic	Staff (n = 13)	Educational (n = 20)	Community/ freelance (n = 12)	Video remote (n = 31)	Video relay (n = 41)	Other (n = 3)	Total (N = 120)	*P*
Age, mean (SEM), y	42.2 (3.1)	44.6 (2.3)	42.0 (2.6)	45.4 (2.0)	49.8 (1.5)	57.3 (5.9)	46.4 (1.0)	.001
**Hearing status**								
Deaf	0	0	0	0	0	0	0	.07
Hard-of-hearing	0	0	1 (8.3)	0	0	0	1 (0.8)	
Hearing	13 (100.0)	20 (100.0)	11 (91.7)	31 (100.0)	41 (100.0)	3 (100.0)	119 (99.2)	
**Sex/gender**								
Female	10 (76.9)	19 (95.0)	10 (83.3)	25 (80.6)	31 (75.6)	3 (100.0)	98 (81.7)	.65
Male	2 (15.4)	1 (5.0)	1 (8.3)	5 (16.1)	9 (22.0)	0	18 (15.0)	
Trans male/trans man	0	0	1 (8.3)	0	0	0	1 (0.8)	
Trans female/trans woman	0	0	0	0	0	0	0	
Gender queer/gender nonconforming	1 (7.7)	0	0	0	1 (2.4)	0	2 (1.7)	
Prefer not to say	0	0	0	1 (3.2)	0	0	1 (0.8)	
**Race**								
American Indian or Alaska Native	0	0	0	0	0	0	0	.69
Asian	0	1 (5.0)	0	1 (3.2)	0	0	2 (1.7)	
Black or African American	1 (7.7)	0	0	1 (3.2)	1 (2.4)	0	3 (2.5)	
Hispanic or Latino	1 (7.7)	0	0	0	2 (4.9)	0	3 (2.5)	
Multiracial	1 (7.7)	0	1 (8.3)	0	1 (2.4)	0	3 (2.5)	
Native Hawaiian or Other Pacific Islander	0	0	1 (8.3)	0	0	0	1 (0.8)	
Other	0	0	1 (8.3)	2 (6.5)	0	0	3 (2.5)	
Prefer not to say	0	1 (5.0)	0	1 (3.2)	1 (2.4)	0	3 (2.5)	
White	10 (76.9)	18 (90.0)	9 (75.0)	26 (83.9)	36 (87.8)	3 (100.0)	102 (85.0)	
**Education**								
Less than a high school diploma	0	0	0	0	0	0	0	.71
High school diploma or equivalent	0	0	0	1 (3.2)	2 (4.9)	0	3 (2.5)	
Some college, no degree	2 (15.4)	0	0	2 (6.5)	4 (9.8)	0	8 (6.7)	
Associate degree	1 (7.7)	3 (15.0)	1 (8.3)	5 (16.1)	9 (22.0)	0	19 (15.8)	
Bachelor’s degree	9 (69.2)	11 (55.0)	6 (50.0)	11 (35.5)	11 (26.8)	1 (33.3)	49 (40.8)	
Master’s degree	1 (7.7)	6 (30.0)	5 (41.7)	11 (35.5)	14 (34.1)	1 (33.3)	38 (31.7)	
Professional degree (DDS, DPT, DVM, MD, PharmD)	0	0	0	0	0	1 (33.3)	1 (0.8)	
Doctorate degree	0	0	0	0	0	0	0	
Prefer not to say	0	0	0	1 (3.2)	1 (2.4)	0	2 (1.7)	

Abbreviation: DDS, Doctor of Dental Surgery; DPT, Doctor of Physical Therapy; DVM, Doctor of Veterinary Medicine; MD, Doctor of Medicine; PharmD, Doctor of Pharmacy; SEM, standard error of the mean.

a All values expressed as number (%), unless otherwise indicated.

## Results

### Participants

We surveyed 120 certified sign language interpreters across the US, Canada, and England (Table 2). The overall group worked 28.1 (SEM, 0.9) hours per week and was aged 46.4 (SEM, 1.0) years; 81.7% of the sample was female, 99.2% was hearing, and 85% was White. Roughly 73.3% of the sample had an educational level equivalent to a bachelor’s degree or higher. Thirteen participants (10.8%) worked as staff interpreters (33.8 [SEM, 2.2] h/wk), 20 (16.7%) as educational interpreters (29.1 [SEM, 2.3] h/wk), 12 (10.0%) as community/freelance interpreters (22.6 [SEM, 1.9] h/wk), 31 (25.8%) as video remote interpreters (27.5 [SEM, 2.2] h/wk), 41 (34.2%) as video relay interpreters (28.6 [SEM, 1.2] h/wk), and 3 (2.5%) as other (16.7 [SEM, 6.0] h/wk).

**Figure 1:**
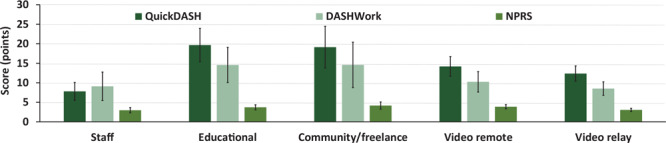
Adjusted mean (SEM) scores on the outcomes for physical health across interpreting settings, including the Quick Disabilities of the Arm, Shoulder, and Hand (QuickDASH), the optional work module on the QuickDASH (DASHWork), and Numeric Pain Rating Scale (NPRS). Abbreviation: SEM, standard error of the mean.

For the physical health measures, the overall adjusted disability score was 13.93 (SEM, 1.43) of 100 and the adjusted work disability score was 10.86 (SEM, 1.59) of 100. The adjusted NPRS was 3.53 (SEM, 0.29) of 10. The greatest to least prevalent body regions of self-reported pain were the shoulder(s) (27.5%), neck (18.3%), wrist(s) or hand(s) (12.5%), low back (8.3%), elbow(s) or forearm(s) (7.5%), mid-back (5.8%), and hip(s) or pelvis (3.3%). “Other” body region pain was reported by 2.5% of respondents, and 14.2% indicated no pain in any body region. No significant differences were found in adjusted disability and work disability scores or NPRS (Figure 1) across settings.

**Table:** 

Interpreting setting	QuickDASH	DASHWork	NPRS
	Mean Score (SEM)		
Staff	7.8 (2.28)	9.1 (3.64)	2.97 (0.68)
Educational	19.66 (4.28)	14.56 (4.5)	3.72 (0.65)
Community/freelance	19.12 (5.36)	14.58 (5.82)	4.17 (0.92)
Video remote	14.19 (2.52)	10.24 (2.62)	3.87 (0.54)
Video relay	12.37 (1.95)	8.52 (1.72)	3.05 (0.39)
χ^2^_4_ [*P* value]	8.614 [.07]	3.948 [.41]	2.959 [.57]

**Figure 2:**
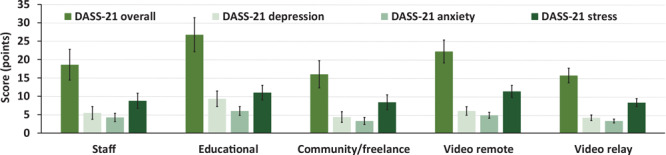
Adjusted mean (SEM) scores on the outcomes for mental health across interpreting settings (mean ± SEM), including the overall Depression, Anxiety, and Stress Scale (DASS-21) and the DASS-21 depression, anxiety, and stress subscales. Abbreviation: SEM, standard error of the mean.

For the DASS-21, participants had an overall adjusted score of 19.44 (SEM, 1.59) out of 126. Scores on the adjusted depression, anxiety, and stress subscales were, respectively, 5.65 (SEM, 0.64), 4.24 (SEM, 0.41), and 9.47 (SEM, 0.81) out of 42 for each subscale. Overall, 45 (37.5%) participants had at least 1 subscale indicating a higher-than-normal level (6 staff, 9 educational, 4 community/freelance, 14 video remote, and 12 video relay). Twenty-one respondents scored above the norm on 1 subscale, 17 scored above the norm on 2 subscales, and 7 scored above the norm on all 3 subscales. Twenty-seven (22.5%) respondents (4 staff, 7 educational, 2 community/freelance, 7 video remote, and 7 video relay) were above the norm for depression: 12 were mild, 8 moderate, 4 severe, and 3 extremely severe. Twenty (16.7%) respondents (2 staff, 4 educational, 1 community/freelance, 7 video remote, and 6 video relay), were above the norm for anxiety: 7 were mild, 10 moderate, 1 severe, and 2 extremely severe. Twenty-nine (24.2%) respondents (4 staff, 7 educational, 3 community/freelance, 9 video remote, and 6 video relay) were above the norm for stress: 14 were mild, 9 moderate, 5 severe, and 1 extremely severe. Twenty-two (18.3%) respondents (3 staff, 6 educational, 2 community/freelance, 7 video remote, and 4 video relay) were above the norm on the overall DASS-21 score. No significant differences were found in the adjusted overall DASS-21 or DASS-21 depression, anxiety, and stress subscale scores (Figure 2) across settings.

**Table:** 

Interpreting setting	Overall	Depression	Anxiety	Stress
	Mean score (SEM)			
Staff	18.59 (4.19)	5.48 (1.72)	4.26 (1.13)	8.78 (2.07)
Educational	26.74 (4.63)	9.33 (2.12)	6.02 (1.19)	11.00 (1.99)
Community/freelance	15.99 (3.70)	4.36 (1.45)	3.29 (0.96)	8.39 (2.01)
Video remote	22.19 (3.11)	6.02 (1.16)	4.83 (0.80)	11.34 (1.64)
Video relay	15.65 (1.98)	4.16 (0.76)	3.29 (0.52)	8.27 (1.09)
χ^2^_4_ [*P* value]	8.072 [.09]	8.407 [.08]	7.231 [.12]	3.681 [.45]

Regarding correlations between the surveyed outcome measures, all mental health measures were positively associated with one another (Table 3), and all physical health measures were positively associated with one another. The QuickDASH was not associated with the DASS-21 depression subscale; otherwise, all physical with mental health measures were positively associated.

**Table 3:** Correlation Matrix of Outcome Measures, Study of the Physical and Mental Health of Sign Language Interpreters in the US, Canada, and England (N = 120) Working Remotely During the COVID-19 Pandemic, Rochester, New York, March–September 2021

Variable	DASS-21 (depression)	DASS-21 (anxiety)	DASS-21 (stress)	DASS-21 (overall)	QuickDASH	DASHWork	NPRS
**DASS-21 (depression)**							
Spearman ρ	1.00	0.313	0.704	0.840	0.149	0.270	0.265
*P* value	—	.001	.001	.001	.11	.003	.004
**DASS-21 (anxiety)**							
Spearman ρ	0.313	1.00	0.425	0.599	0.36	0.347	0.341
*P* value	.001	—	.001	.001	.001	.001	.001
**DASS-21 (stress)**							
Spearman ρ	0.704	0.425	1.00	0.931	0.223	0.387	0.386
*P* value	.001	.001	—	.001	.02	.001	.001
**DASS-21 (overall)**							
Spearman ρ	0.840	0.599	0.931	1.00	0.254	0.393	0.398
*P* value	.001	.001	.001	—	.006	.001	.001
**QuickDASH**							
Spearman ρ	0.149	0.336	0.223	0.254	1.00	0.651	0.636
*P* value	.11	.001	.02	.006	—	.001	.001
**DASHWork**							
Spearman ρ	0.270	0.347	0.387	0.393	0.651	1.00	0.619
*P* value	.003	.001	.001	.001	.001	—	.001
**NPRS**							
Spearman ρ	0.265	0.341	0.386	0.398	0.636	0.619	1.00
*P* value	.004	.001	.001	.001	.001	.001	—

Abbreviations: — , not applicable; DASS-21, Depression, Anxiety, and Stress Scale; DASHWork, optional work module of QuickDASH; NPRS, Numeric Pain Rating Scale; QuickDASH, shortened version of the Disabilities of the Arm, Shoulder, and Hand.

## Discussion

We found that, although the overall means for the mental health measures were considered within normal limits, roughly 38% of respondents scored above the norm on at least 1 subscale. Community/freelance, staff, and video relay interpreting settings consistently had the lowest number of interpreters with higher-than-normal levels for depression, anxiety, and stress, indicating fewer mental health concerns compared with educational and video remote settings when working remotely. The positive association between outcomes for physical with mental health supported this study’s hypothesis and was consistent with the previous literature studying onsite interpreters (2–4). In contrast to our study’s hypothesis, we found no significant differences across interpreting settings. Instead, our study served a heuristic purpose in identifying trends and possible areas for further research.

The body region with the highest prevalence of pain and pain intensities reported while working remotely were slightly different when compared with past reports, likely due to the difference in work conditions. Roman and Samar found that video relay interpreters working onsite in call centers reported the highest prevalence of pain in the cervical spine (34%) (18), whereas we found the highest prevalence in the shoulder(s) (27.5%). Roman and Samar also found that 18% of interpreters had no report of musculoskeletal pain, which was similar to the 14% that we identified. Although we found no significant differences across interpreting settings in pain intensities, the reported mean NPRS was higher than the baseline pain reported from signers with pain (19) and comparable with the postinterpreting pain intensity from interpreters with pain (20), indicating an elevated baseline pain rating for interpreters when working remotely.

Our study had limitations. Our ability to detect causation between remote interpreting with physical and mental health was limited by the cross-sectional design. During the pandemic, adults in the US demonstrated a threefold increased prevalence of mental health concerns compared with prepandemic estimates (21). Roughly 24.3% of adults had depressive disorder symptoms and 25.5% expressed symptoms of anxiety (22) compared with the 22.5% of the interpreters in this study who had higher-than-normal levels for depression and 16.7% with higher-than-normal levels for anxiety. The nonrandomized sampling methodology in our work did not draw a representative sample, thus comparisons to the general population are limited. Additionally, there was likely a response or self-selection bias from the interpreters who chose to complete the survey. Participants with a history of physical or mental health pathologies secondary to activities other than interpreting were not excluded from the study, and findings were not cross-referenced with health information before working remotely. Although the DASS-21 asked respondents to “please read each statement and click on how much the statement applied to you over the past week while working remotely,” it was difficult to discern whether these adverse mental health symptoms presented secondary to the condition of remote interpreting, the mass trauma from the pandemic, both, or otherwise.

There were several other limitations to this study. The field of sign language interpreting has a preponderance of White, hearing, female workers (23,24). More research is needed on the remote working experience of interpreters who represent a broader multicultural perspective. Data on the educational levels of interpreters show that 36%, 18%, and 1.5% have bachelor’s, master’s, and doctoral degrees, respectively (24). Our study had a slightly higher representation of interpreters with master’s degrees (31.7%). Our data were also powered to detect differences in perception of stress across onsite and remote spoken language interpreting (9) and not to determine physical and mental health differences across interpreting settings. However, the use of separate generalized linear models while adjusting for distribution and the notable demographic difference in age across settings was robust. The descriptively identified trends should help to guide further research with larger sample sizes.

This work provided additional insight on the physical and mental health of interpreters working remotely. Results suggested that shoulder pain was most prevalent and baseline pain levels were elevated. Associations between disability, work disability, and pain with mental health warrant a holistic approach in the clinical treatment and research of this essential worker population. Educational and community/freelance interpreters trended toward greater physical health concerns. Although depression, anxiety, and stress for the overall sample were within normal limits, educational and video remote interpreters trended toward greater mental health concerns. Because communication access for deaf communities promotes equity and inclusion, more work is needed to further understand the impact of changing work conditions because of the pandemic on the occupational health of sign language interpreters.

Our work fills a gap in addressing needs of the particularly vulnerable (and under-researched) populations of sign language interpreters and the deaf and hard-of-hearing population that relies on them. Imagine if the availability of interpreters was limited because of adverse mental health to process calls via 9-1-1 for emergency services. A multitude of interpreting scenarios highlight the importance of this research to protect and promote the livelihood of interpreters and ensure the continued availability of interpreting services to hearing and deaf consumers. Communication access is a social determinant of health for deaf communities (25). Primary care providers were less likely to deliver preventive services to patients who are deaf sign language users in the absence of an interpreter (13,26), which has implications for the long-term health outcomes and prevention of chronic disease for deaf communities. Sign language interpreters serve a critical role in addressing the language barriers that have resulted in health inequities. The Centers for Medicare and Medicaid Services suggest that organizations would benefit from having a communication access plan and that reasonable accommodations, including providing qualified interpreters, will ensure effective communication with individuals who are deaf and hard of hearing (27). The National Association of the Deaf recommends the procurement of qualified sign language interpreters in their position statements on best practices for effective communication during emergency press conferences (28) and health care access (29). We hope this work will serve to increase awareness of how the adverse occupational health of sign language interpreters can have public health implications. Findings will help guide maximum variation sampling for qualitative data collection in interpreting setting-specific focus groups and supplement examination of the determinants of implementation behavior (30) across settings upon transitioning from onsite to remote interpreting in future work.
